# Supplementary material for: EOSnet: Embedded Overlap Structures for Graph Neural Networks in Predicting Material Properties
Source: J Phys Chem Lett. 2025 Jan 11;16(3):717–24. doi: 10.1021/acs.jpclett.4c03179 (PMC12333346; doi:10.1021/acs.jpclett.4c03179)
Supplement: Supplementary file 1 [file jz4c03179_si_001.pdf]

Supporting Information: EOSnet: Embedded Overlap Structures for Graph Neural Networks in Predicting Material Properties

Shuo Tao and Li Zhu*

Department of Physics, Rutgers University, Newark, NJ 07102, United States of America

E-mail: li.zhu@rutgers.edu

Elements of Gaussian Overlap Matrix

The Gaussian Overlap Matrix (GOM) is a key component in the EOSnet model, which is used to encode the local atomic environment of each atom in the unit cell. In addition to the definition of the GOM, we also provide the expressions for some of the GOM overlap elements:

$$\begin{aligned}
\left\langle \phi_i^s \middle| \phi_j^s \right\rangle &= S_{ij} = S_{ji} = \left(\frac{2\sqrt{\alpha_i \alpha_j}}{\alpha_i + \alpha_j} \right)^{3/2} \exp\left[\frac{-\alpha_i \alpha_j}{\alpha_i + \alpha_j} r_{ij}^2 \right] \\
\left\langle \phi_i^{p_x} \middle| \phi_j^s \right\rangle &= \frac{1}{\sqrt{\alpha_i}} \frac{\partial S_{ij}}{\partial x_i} = -\left(\frac{2\sqrt{\alpha_i}\alpha_j}{\alpha_i + \alpha_j} \right) (x_i - x_j) S_{ij} \\
\left\langle \phi_i^{p_x} \mid \phi_j^{p_{x'}} \right\rangle &= \left(\frac{2\sqrt{\alpha_i \alpha_j}}{\alpha_i + \alpha_j} \right) S_{ij} \left[\delta_{x,x'} - \frac{2\alpha_i \alpha_j}{\alpha_i + \alpha_j} (x_i - x_j)\left(x_i' - x_j'\right) \right]
\end{aligned} \tag{1}$$

In this work, we explored a range of cutoff radii and found that a cutoff of approximately

8 Ågenerally provides a good balance between accuracy and computational cost. With this cutoff, we considered the first 66 neighboring atoms to construct the Gaussian Overlap Matrix (GOM). Regarding orbital choices, we tested both the inclusion of only s-orbitals and the combination of *s*- and *p*-orbitals. We observed only minor differences in performance. For computational efficiency, we chose to use *s*-orbitals only in the current work. In future studies, we plan to conduct more extensive tests to investigate the impact of different cutoff values and orbital selections in greater detail.

Validation of Fingerprint Integration in GNN

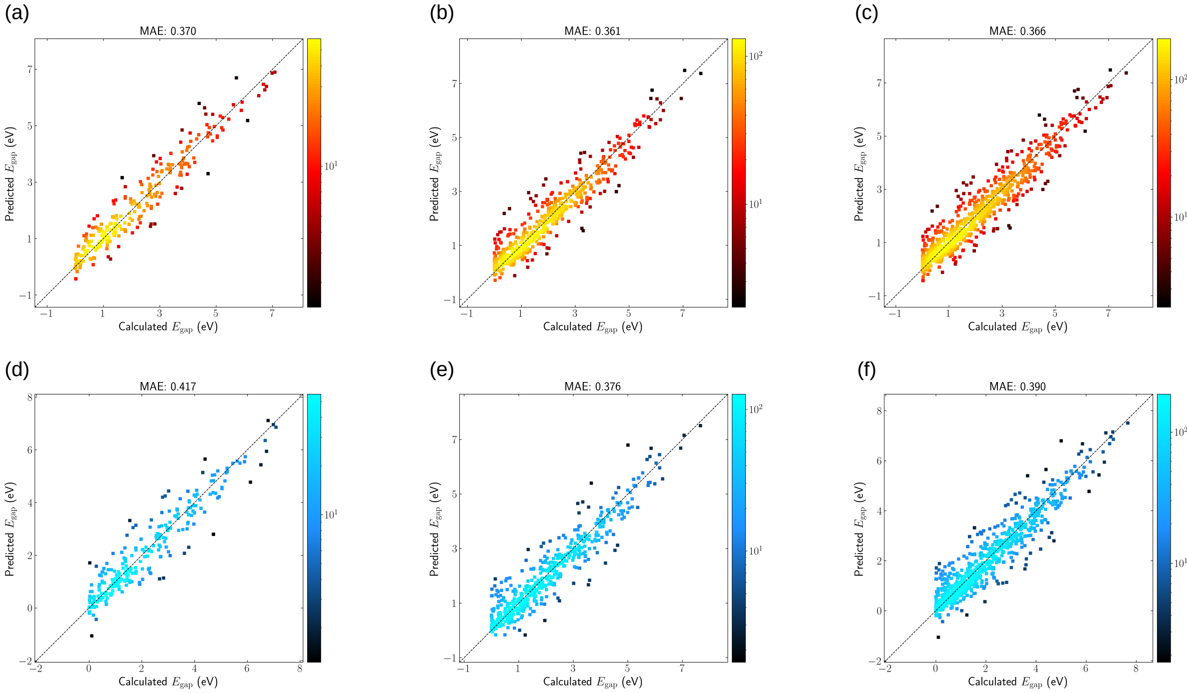

Figure S1: MAEs and parity plots for GNN models with (red) and without (blue) including Fingerprints as node features for: (a), (d) direct-gap semiconductors, (b), (e) indirect-gap semiconductors, and (c), (f) all semiconductors combined. The data comprises 2,650 direct-gap and 6,308 indirect-gap semiconductors, totaling 8,958 non-metallic entries from the 19,393 Material Projects dataset used in the main text.

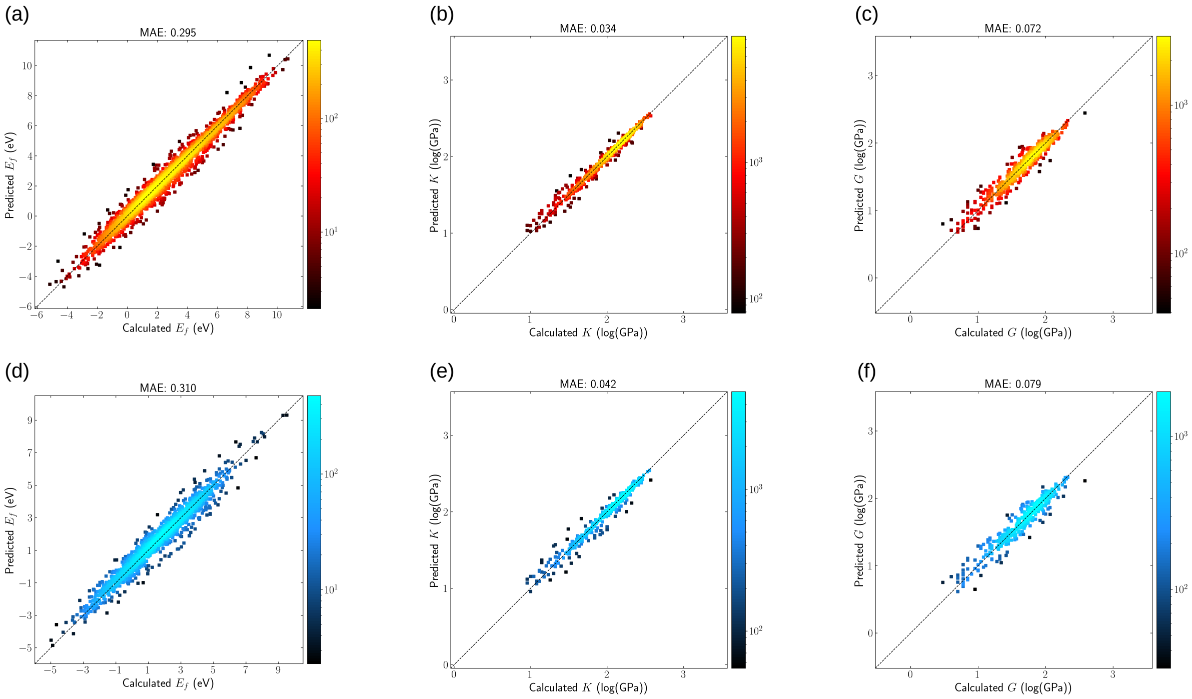

Figure S2: MAEs and parity plots for GNN models with (red) and without (blue) Fingerprints as node features for: (a), (d) Fermi energy, (b), (e) bulk modulus, and (c), (f) shear modulus. The datasets consist of 27,293 Fermi energy entries and 5,000 entries each for bulk and shear moduli from the Materials Project.

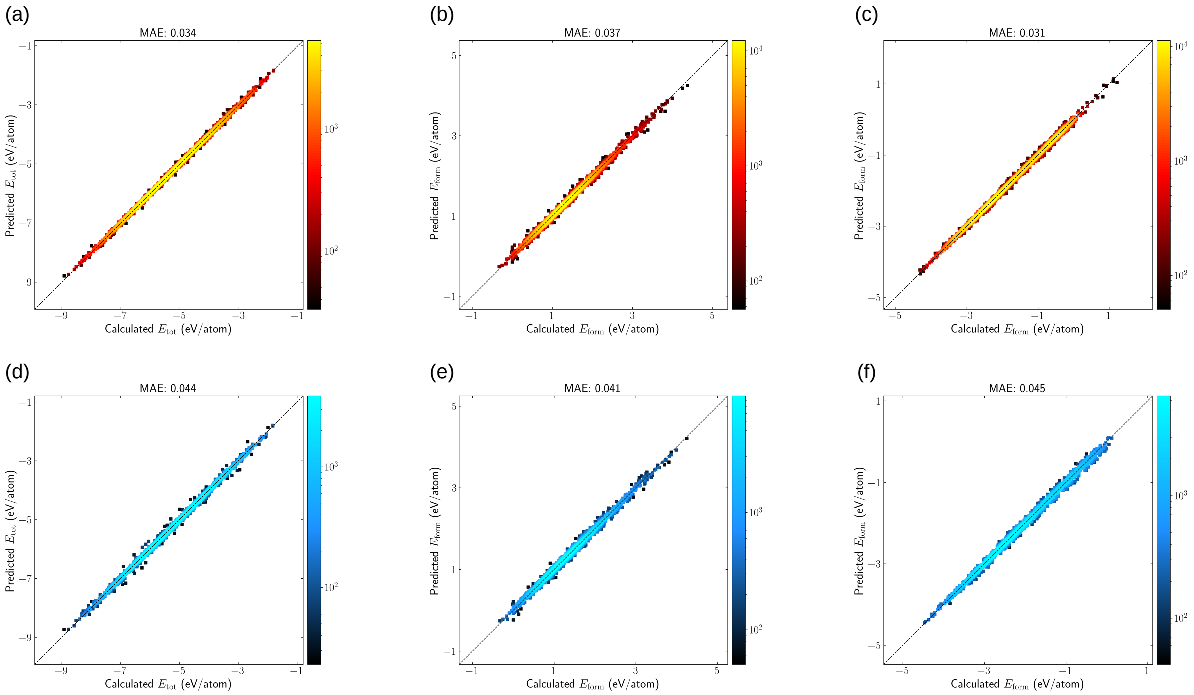

Figure S3: MAEs and parity plots for GNN models with (red) and without (blue) Fingerprints as node features for: (a), (d) total energy from 19,364 entries in the ASE cubic perovskite database, (b), (e) formation energy from 19,364 entries in ASE and (c), (f) formation energy from 27,293 entries in the Materials Project.

Comparing Results for Different Size of Dataset

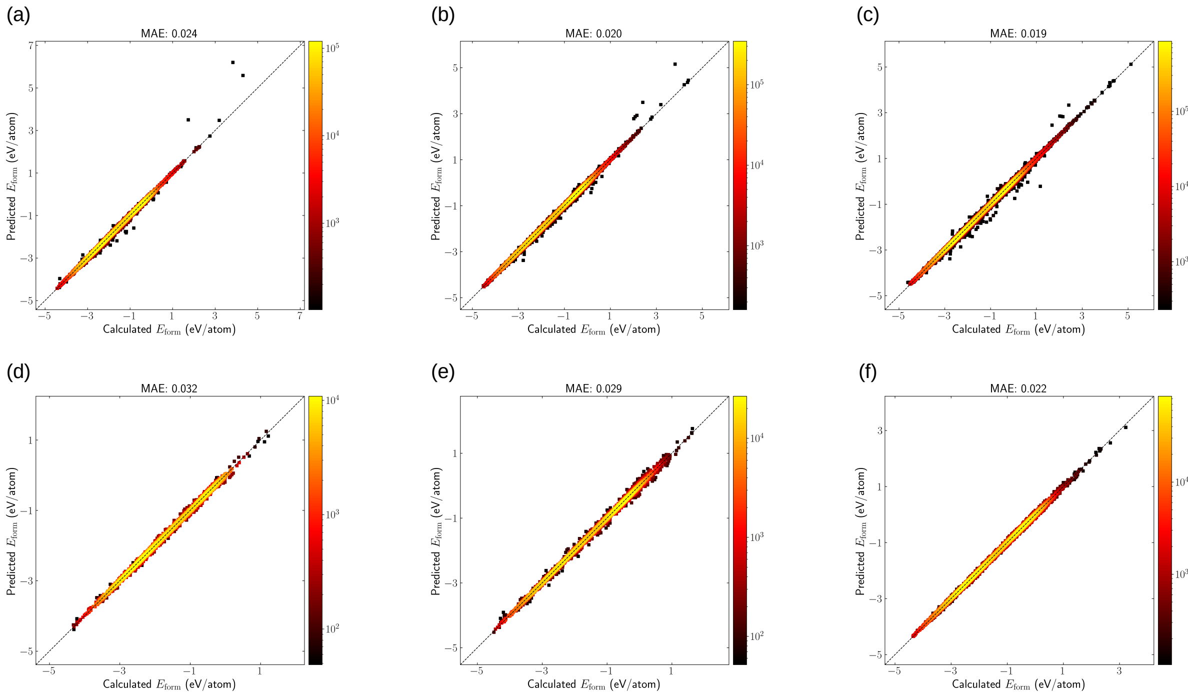

Figure S4: MAEs and parity plots of formation energy (eV/atom) for different size of Materials Project dataset: Training set plots for (a) small size (21,834), (b) medium size (40,523) and (c) large size (104,992) MP dataset, respectively. Testing set plots for (d) small size (2,729), (e) medium size (5,065) and (f) large size (13,124) MP dataset, respectively.

And following are the histograms for distribution of number of elements per unit cell

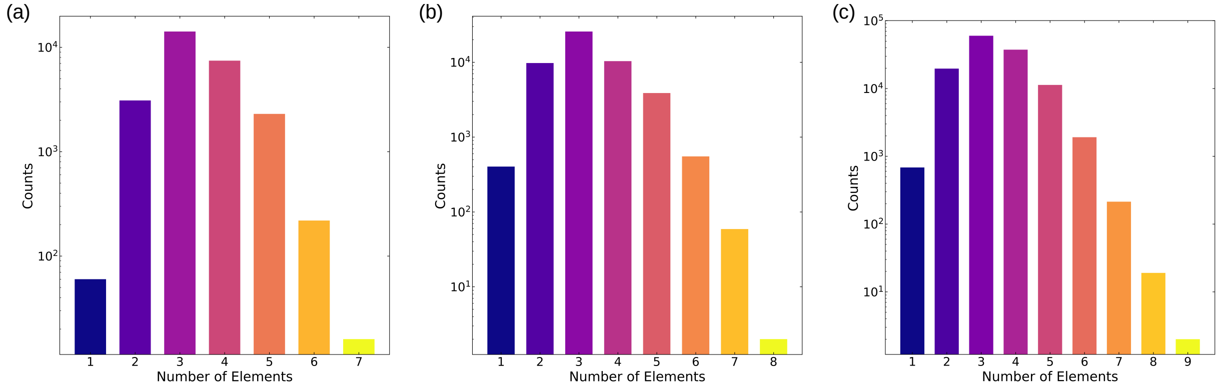

Figure S5: Histogram for distribution of number of elements per unit for (a) small size (27,293), (b) medium size (50,654) and (c) large size (131,240) MP dataset before train-validation-test splitting.

List of Parameters

Table S1: A list of parameters for model configuration in this work.

**Parameter**	**Value**
Cutoff radius (Å)	8.0
Maximum number of GOM elements	256
l_{max} (0 for s orbital only)	0
Maximum number of neighbors	12
Number of convolutional layers	3
Number of hidden atom features in convolutional layers	64
Number of hidden features after pooling	128
Number of hidden layer	1
Batch size	64, 128, 256
Normalization type	Batch normalization
Total training epochs	500
Initial learning rate	0.001
Warm-up epochs	20
LR milestone	[100, 200, 400]
